# Divergent Control of Two Type VI Secretion Systems by RpoN in *Pseudomonas aeruginosa*

**DOI:** 10.1371/journal.pone.0076030

**Published:** 2013-10-21

**Authors:** Thibault G. Sana, Chantal Soscia, Céline M. Tonglet, Steve Garvis, Sophie Bleves

**Affiliations:** Laboratoire d’Ingénierie des Systèmes Macromoléculaires (UMR7255), CNRS & Aix-Marseille Univ, Marseille, France; Institut Pasteur, France

## Abstract

Three Type VI Secretion System (T6SS) loci called H1- to H3-T6SS coexist in *Pseudomonas aeruginosa*. H1-T6SS targets prokaryotic cells whereas H2-T6SS mediates interactions with both eukaryotic and prokaryotic host cells. Little is known about the third system, except that it may be connected to H2-T6SS during the host infection. Here we show that H3-T6SS is required for *P. aeruginosa* PAO1 virulence in the worm model. We demonstrate that the two putative H3-T6SS operons, called “left” and “right”, are coregulated with *H2-T6SS* by the Las and Rhl Quorum Sensing systems. Interestingly, the RpoN σ54 factor has divergent effects on the three operons. As for many T6SSs, RpoN activates the expression of H3-T6SS left. However, RpoN unexpectedly represses the expression of H3-T6SS right and also H2-T6SS. Sfa2 and Sfa3 are putative enhancer binding proteins encoded on H2-T6SS and H3-T6SS left. In other T6SSs EBPs can act as σ54 activators to promote T6SS transcription. Strikingly, we found that the RpoN effects of H3-T6SS are Sfa-independent while the RpoN mediated repression of H2-T6SS is Sfa2-dependent. This is the first example of RpoN repression of a T6SS being mediated by a T6SS-encoded EBP.

## Introduction

Protein secretion is an essential for host colonization by pathogenic bacteria. Multiple systems have evolved in order to secrete proteins into the extracellular medium or directly into target cells [1]. The most recently described system, the Type Six Secretion System (T6SS), was first discovered in *Vibrio cholerae* [2] and in *Pseudomonas aeruginosa* [3]. A unique feature of T6SSs is their capacity to deliver toxic proteins into eukaryotic host cells as well as into bacteria [4], [5]. These systems were originally thought of as virulence determinants towards eukaryotic host cells [6], [7], although now they have also been shown to be unambiguously involved in interbacterial interactions and competition [8]. Nevertheless, the anti-prokaryotic T6SSs may also facilitate the colonization of specific niches where pathogens can then express virulence towards eukaryotic cells. Another striking feature of T6SSs is that multiple distinct T6SS loci are often present in a single genome. For example, the genomes of *Burkholderia pseudomallei* [9], [10] and *Yersinia pseudotuberculosis* [11] harbor six and four T6SSs respectively. The various T6SS within a single strain may serve different functions and/or be differentially regulated. In *Burkholderia thailandensis*, the T6SS-5 was shown to be required for virulence in a murine melioidosis model, while inactivation of T6SS-1 rendered *B. thailandensis* more susceptible to contact with other bacteria [12].

The genome of the *P. aeruginosa* contains three T6SS loci, called H1- to H3-T6SS [3], [7]. H1-T6SS has been widely studied and was shown to deliver three bacteriolytic toxins to the periplasm of target bacteria [8], [13]. It may give *P. aeruginosa* a survival benefit in a multi-bacterial environment. However, less is known about the two other T6SSs. The H2-T6SS locus of the PAO1 strain of *P. aeruginosa* promotes bacterial internalization into epithelial cells, and indeed plays a role in virulence in the worm model [14]. H2-T6SS also mediates interbacterial competition through Tle5/PldA [15], a phospholipase D which was previously shown to contribute to *P. aeruginosa* persistence in a chronic pulmonary infection model [16]. As with many *P. aeruginosa* virulence factors, the H2-T6SS machinery is controlled by Quorum-Sensing (QS) and iron availability in the environment [14]. H2-T6SS and H3-T6SS loci in the PA14 strain of *P. aeruginosa* differ from their counterparts in the PAO1 strain by the absence of three and one putative effector genes [17]. PA14 H2-T6SS and H3-T6SS are both required for virulence in the plant model *Arabidopsis thaliana*. In a mouse model of acute infection, while a H2-T6SS mutant was affected, a H3-T6SS mutant was as virulent as the Wild-Type (WT) strain. Interestingly, the double H2- and H3-T6SS mutant exhibited dramatically reduced virulence, this suggesting compensation *in vivo* between the two systems [17].

In this study, we aimed at determining whether the H2-T6SS and H3-T6SS loci of PAO1 are related at a transcriptional level. We observed that, like H2-T6SS, the two H3-T6SS gene clusters are activated by Quorum Sensing, but are under divergent control by RpoN.

## Materials and Methods

### Bacterial Strains, Plasmids, and Growth Conditions

The bacterial strains and plasmids used in this study are described in Table 1. LB and TSB broths and agar were used for the growth of *P. aeruginosa* and *Escherichia coli* strains at 37°C. Cultures were inoculated at an optical density at 600 nm (OD_600_) of 0.1 with overnight cultures, and strains were grown at 30, 37 or 42°C with aeration in TSB. Recombinant plasmids were introduced into *P. aeruginosa* using the conjugative properties of pRK2013 (Table 1) or by electroporation. *Pseudomonas* transconjugants were selected on *Pseudomonas* isolation agar (PIA, Difco Laboratories) supplemented with appropriate antibiotics. The antibiotic concentrations were as follows: for *E. coli*, ampicillin (50 µg ml^–1^), kanamycin (25 µg ml^–1^), tetracycline (15 µg ml^–1^), gentamicin (10 µg ml^–1^); for *P. aeruginosa*, tetracycline (200 µg ml^–1^ for plates or 50 µg ml^–1^ for liquid growth), gentamicin (50 µg ml^–1^), carbenicillin (500 µg ml^–1^).

**Table 1:** Strains, plasmids and oligonucleotides used in this study.

Strain, plasmid oroligonucleotide	Genotype, descriptionor sequence	Source and/or reference
***E. coli*** ** strains**		
TG1	*supE,* Δ*(mcrB-hsdSM)5, thi-1,* Δ*(lac-proAB),* F′ (*traD36, proAB+, lacI_q_, lacZ*Δ*M15*)	Laboratory collection
CC118(λpir)	(λ*pir*) Δ(*ara-leu*), *araD,* Δ*lacX74, galE, galK, phoA-20, thi-1, rpsE, rpoB,* *Arg*(Am), *recA1,* Rfr (λ*pir*)	[42]
TOP10F’	F- *mcrA* Δ*(mrr-hsdRMS-mcrBC) φ80lacZ*Δ*M15* Δ*lacX74 nupG recA1 araD139* Δ(*ara-leu*)7697*galE15 galK16 rpsL*(Str^R^) *endA1* λ^-^	Laboratory collection
***P. aeruginosa strains***		
PAO1	Wild-type, prototroph, *chl*-2	B. Holloway
PAO1Z	Promoterless *lacZ* gene integrated at ctx att site in PAO1	This work
PAO1TS2	*H2-T6SS* promoter integrated at ctx att site in PAO1	[14]
PAO1TS19	*H3-T6SS left* promoter integrated at ctx att site in PAO1	This work
PAO1TS20	*H3-T6SS right* promoter integrated at ctx att site in PAO1	This work
PAO1Δ*clpV2*	*clpV2* deletion mutant	[14]
PAO1Δ*clpV3*	*clpV3* deletion mutant	[20]
PAO1Δ*clpV2*Δ*clpV3*	*clpV2* and *clpV3* deletion mutant	This work
PAO6358	PAO1 *rpoN* deletion mutant	[28]
PAO6360	PAO1Δ*rpoN att* Tn7*::rpoN^+^* GmR	[28]
PAO1R	*lasR* mutant of PAO1, CbR	[24]
PDO100	*rhlI* mutant of PAO1, HgR	[25]
PAO6358TS2	*H2-T6SS* promoter integrated at ctx att site in PAO1Δ*rpoN*	This work
PAO6358TS19	*H3-T6SS left* promoter integrated at ctx att site in PAO1Δ*rpoN*	This work
PAO6358TS20	*H3-T6SS right* promoter integrated at ctx att site in PAO1Δ*rpoN*	This work
PAO6360TS2	*H2-T6SS* promoter integrated at ctx att site in PAO1Δ*rpoN att* Tn7*::rpoN^+^*	This work
PAO6360TS19	*H3-T6SS left* promoter integrated at ctx att site in PAO1Δ*rpoN att* Tn7*::rpoN^+^*	This work
PAO6360TS20	*H3-T6SS right* promoter integrated at ctx att site in PAO1Δ*rpoN att* Tn7*::rpoN^+^*	This work
PAORTS19	*H3-T6SS left* promoter integrated at ctx att site in PAOR	This work
PAORTS20	*H3-T6SS right* promoter integrated at ctx att site in PAOR	This work
PDO100TS19	*H3-T6SS left* promoter integrated at ctx att site in PDO100	This work
PDO100TS20	*H3-T6SS right* promoter integrated at ctx att site in PDO100	This work
PAO1*sfa2*	*sfa2* mutant of PAO1, CbR	This work
PAO *sfa2*TS2	*H2-T6SS* promoter integrated at ctx att site in PAO1*sfa2*	This work
PAO *sfa2*TS19	*H3-T6SS left* promoter integrated at ctx att site in PAO1*sfa2*	This work
PAO *sfa2*TS20	*H3-T6SS right* promoter integrated at ctx att site in PAO1*sfa2*	This work
PAO1*sfa3*	*sfa3* mutant of PAO1, CbR	This work
PAO *sfa3*TS2	*H2-T6SS* promoter integrated at ctx att site in PAO1*sfa3*	This work
PAO *sfa3*TS19	*H3-T6SS left* promoter integrated at ctx att site in PAO1*sfa3*	This work
PAO *sfa3*TS20	*H3-T6SS right* promoter integrated at ctx att site in PAO1*sfa3S*	This work
**Plasmids**		
pCR2.1	TA cloning, *lacZ*a, ColE1, f1 ori, ApR KmR	Invitrogen
pMini-CTX::*lacZ*	Ω-FRT-attP-MCS, ori, int, oriT, TcR	[18]
pMP220	Broad host-range *lacZ* transcriptional fusion, TcR	Laboratory collection
pRK2013	Tra+, Mob+, ColE1, KmR	Laboratory collection
pKNG101	oriR6K, mobRK2, sacBR+, SmR (suicide vector)	[43]
pJN105	GmR, *araC*-pBAD	[44]
pTS2	722 bp upstream region of *H2-T6SS* in pMini-CTX::lacZ	[14], [25]
pTS12	486 bp upstream region of *H3-T6SS left* in pCR2.1	This work
pTS13	494 bp upstream region of *H2-T6SS right* in pCR2.1	This work
pTS19	486 bp upstream region of *H3-T6SS left* in pMini-CTX::lacZ	This work
pTS20	494 bp upstream region of *H2-T6SS right* in pMini-CTX::lacZ	This work
pTS25	*sfa2* gene in pCR2.1	This work
pTS27	500 bp upstream and 500 bp downstream *clpV2* in pKNG101	[14]
pSBC52	*sfa2* gene in pJN105	This work
pSBC56	490 bp internal fragment of *sfa2* cloned in pCR2.1	This work
pSBC57	511 bp internal fragment of *sfa3* cloned in pCR2.1	This work
pMAL.R	P*lasR-lacZ* transcriptional fusion in pMP220	[14], [24]
pMAL.V	P*rhlR-lacZ* transcriptional fusion in pMP220	[24]
**Oligonucleotide** ***s***		
TSO15	5′-CCAGGCTCCATACCGCGAACTG-3′	This work
TSO16	5′-GGCGGCTGACTCCGATGCAA-3′	This work
TSO17	5′-TTGCTGTCGTCGCCGCTGAT-3′	This work
TSO18	5′-GGGAGTCCAACGAAAATTTTATTTTGC-3′	This work
TSO39	5′-ATGTCCGTCATCACCCATCCCCACG-3′	This work
TSO40	5′-TCAGGTCCGGGGATCGCCGAAATG-3′	This work
TSO41	5′-ATGTTCAGCCGCGTACCGCAACC-3′	This work
TSO42	5′-TCACTTGCCCACCAGCGAGACCACG-3′	This work
TSO45	5′-CATGCGACGCTGGGCGAGCACG-3′	This work
TSO46	5′-AATCTATGGGTTCCTGGGGCAAGATGGG-3′	This work
TSO118	5′-ACCTGCAGGGAT TC CCCATCC-3′	This work
TSO119	5′-TGCAACACG CGCAACAGCTTGG-3′	This work
TSO120	5′-TCGTCGCGGTCAACTGCGGTGC-3′	This work
TSO121	5′-TTCTCCAGCTCG CGGATATTGC-3′	This work
OA14	5′-GGAAAGCTTTTCGCCCTCGTCGGATTG-3′	[14]
OA17	5′-AAAGAATTCGAGGCGTTGCAGCAGATG-3′	[14]

### *lacZ* Reporter Fusion and β-galactosidase Assay

The *H3-T6SS left-lac* *Z* and *H3-T6SS right-lac* *Z* transcriptional fusions were constructed by PCR amplification of respectively 486 and 494 bp upstream DNA region from the *lip3* or *hsiB3* gene by using TSO15/TSO16 and TSO17/TSO18 primers (Table 1). PCR amplification products were directly cloned into the pMini-CTX::lacZ vector [18], yielding pTS12 and pTS13, in pCR2.1, and pTS19 and pTS20 in MiniCTX*-‘lac* *Z*. Nucleotide sequences were verified by sequencing (GATC). The promoter fragment was integrated at the CTX phage attachment site in PAO1 and isogenic mutants using established protocols [18].

Overnight culture, grown in TSB, was diluted in TSB to OD_600_ = 0.1. Growth and ß-galactosidase activity were monitored by harvesting samples at different time intervals. ß-galactosidase activity was measured according Miller [19], based on o-nitrophenyl-β-D-galactopyranoside hydrolysis. ß-galactosidase activities were expressed in Miller units.

### Construction of the Δ*clpV2*Δ*cplV3* Mutant

To generate the Δ*clpV2cplV3* mutant, the pTS27 mutator plasmid [14] was mobilized in the *P. aeruginosa* strain PAO1Δ*clpV3* [20]. Mutants which had undergone a double recombination event, resulting in the non-polar deletion of the *clpV2* gene, were verified by PCR with the primers OA14 and OA17 that flank *clpV2*.

### Construction of *sfa2 and sfa3* Mutants

To generate *sfa2* and *sfa3* mutants, internal fragments of 490 and 511 bp were respectively amplified with TSO118-TSO119 and TSO120-TSO121 and cloned in the pCR2.1, resulting in pSBC56 and pSBC57. The mutator plasmids were electroporated into *P. aeruginosa* PAO1 and the mutant bacteria selected on PIA medium containing Carbenicillin. The insertions were verified by PCR with the primer pairs TSO39-TSO40-TSO45 and TSO41-TSO42-TSO46 that hybridize outside and inside of the *sfa2* and *sfa3* genes.

### *Caenorhabditis elegans* Killing Assay

The slow killing assay was performed as described previously [14]. Each independent assay consisted of three replicates. *E. coli* OP50 was used as a control. L4 to adult stage *C. elegans* were removed from food and placed on unseeded NGM plates for 24 hours at 25°C. 50 worms were then picked onto plates containing overnight grown bacteria. Worms were evaluated for viability on a daily basis. Animal survival was plotted using the PRISM 5.0 program. Survival curves are considered significantly different from the control when P-values are <0.05. Prism calculates survival fractions using the product limit (Kaplan-Meier) method. Prism compares survival curves by two methods: the log-rank test (also called the Mantel-Cox test) and the Gehan-Breslow-Wilcoxon test.

### Statistical Analysis

Paired Student’s *t* tests were performed using Excel software. In the figures, * means P-values ≤0.05, ** ≤0.01, and *** ≤0.001.

## Results

### H2 and H3-T6SS are Both Involved in Virulence Towards *C. elegans*

**Figure 1:**
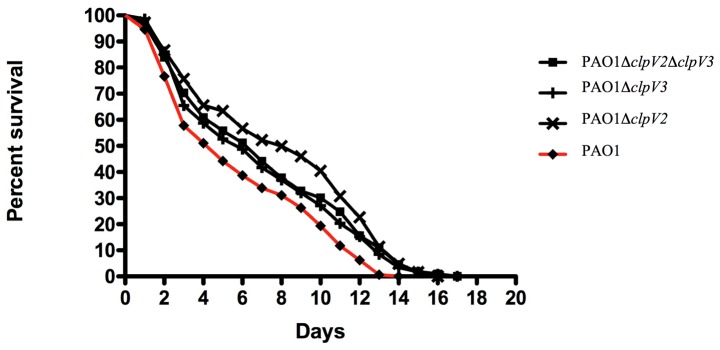
H3-T6SS is required for virulence in *C. elegans*. *C. elegans* was infected with PAO1 and isogenic Δ*clpV2*, Δ*clpV3* or Δ*clpV2*Δ*clpV* **3** mutants. The resulting *C. elegans* survival curve is shown for each strain. Evalue <0.01 (Prism 5 software).

Functional compensation between PA14 H2- and H3-T6SS has been observed in a lung infection model [17]. We previously showed that the H2-T6SS of PAO1 mediates virulence in a worm model [14]. Therefore, we wondered whether the H3-T6SS of PAO1 is also required for virulence. We indeed found decreased virulence in a PAO1 H3-T6SS mutant, which harbors a deletion of the *clpV3* gene (Fig. 1). *clpV3* encodes the ClpV AAA^+^ ATPase [20], a core component of the secretion machinery (Fig. 1). The worms infected with the mutant appeared to die with a 2 day delay when compared to the WT strain. H3-T6SS is thus necessary for killing *C. elegans*, however to a lesser extent than H2-T6SS [14]. In contrast with the findings for PA14 [17], the virulence of the double mutant Δ*clpV2*Δ*clpV3* was not lower than in the single mutants. Both T6SS are thus required for virulence in PAO1.

### *H2-T6SS* and *H3-T6SS* are Coregulated by Quorum-Sensing

**Figure 2:**
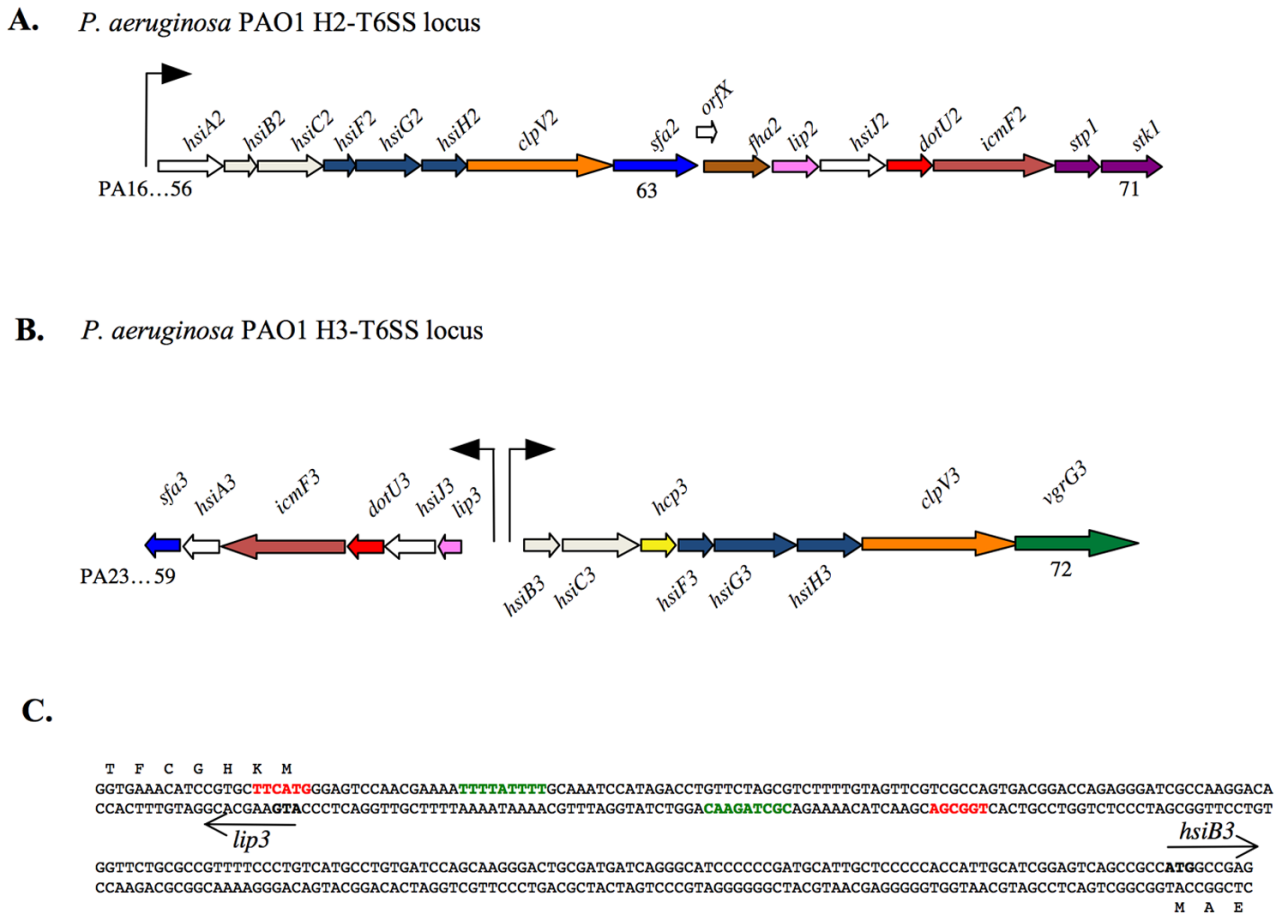
The *P. aeruginosa* PAO1 H2- and H3-T6SS gene clusters. **A**. H2-T6SS is organized in one putative operon (from [14]). **B.** H3-T6SS is organized in two putative operons. The genes are labeled *hsiA3* to *hsiJ3* for the left operon and *hsiB3* to *hsiH3* for the right operon and, where applicable, with the given name, i.e., *clpV3* or *sfa3*. Gene annotation numbers are also indicated (e.g. PA2359). The promoter region of each operon is also shown. **C.** The intergenic sequence between *lip3* and *hsiB3* genes is represented. The –35 box and the –10 box of the σ^70^ promoters predicted by Bprom are highlighted in green and red respectively. The translation initiation codons of *lip3* and *hsiB3* genes are underlined. 486 bp of the left operon upstream region and 494 bp of the right operon upstream region were used for the transcriptional fusions, which are encoded by pTS19 and pTS20 respectively.

**Figure 3:**
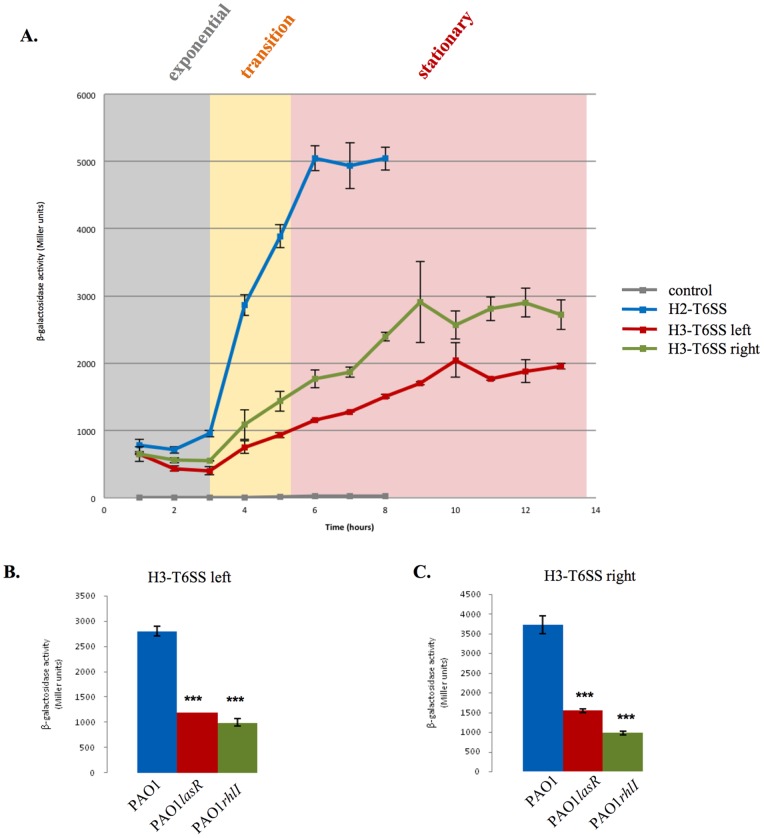
*H2-T6SS* and *H3-T6SS* are coregulated by QS. **(A)** Expression patterns of the *H2-T6SS*, *H3-T6SS* left, and *H3-T6SS* right *lacZ* transcriptional fusions from the WT PAO1 strain (PAO1TS2, PAO1TS19 and PAO1TS20 respectively) and of a control strain (PAO1Z). Expression is given in Miller Units at different time points during growth at 37°C in TSB medium (see Material and Methods). The growth phases are represented here and the growth curves are presented in Fig. S1. Expression of *H3-T6SS* left **(B)** and of *H3-T6SS* right **(C)** is shown in the WT (blue bars) or in QS mutant strains after 4 hours of growth at 37°C. The PAOR background (red bars) is a *lasR* mutant and PDO100 (green bars) a *rhlI* mutant. Each experiment was done in triplicate and independently repeated three times; error bars indicate the standard deviation.

While the *H1-T6SS* is not expressed in a PAO1 WT background [3], [21], *H2-T6SS* expression is controlled by QS in a cell-density dependent manner [14] (Fig. 2A). We therefore investigated the transcriptional regulation of the H3-T6SS gene cluster in PAO1. The H3-T6SS locus is organized into two divergent gene clusters (Fig. 2B), and we analyzed the intergenic DNA region between *lip3* (PA2364) and *hsiB3* (PA2365) for potential regulatory elements (Fig. 2C). The BProm algorithm identified one σ^70^ dependent promoter upstream of the *lip3* gene and another, in the opposite direction, upstream of the *hsiB3* gene (http://linux1.softberry.com/berry.phtml?topic=bprom&group=programs&subgroup=gfindb) (Fig. 2C). To construct chromosomal transcriptional *lac* *Z* fusions, the regions upstream of the ATG of *lip3* and *hsiB3*, the first gene of each of the two H3-T6SS clusters respectively (hereinafter called “left” and “right operons” for simplicity) were fused to *lac* *Z* and then integrated at the CTX phage *attB* site as a single copy on the chromosome, yielding strains PAO1TS19 and PAO1TS20 respectively (Table 1). Strain PAO1Z was similarly constructed by integrating a promoter-less *lac* *Z* gene to serve as a negative control. The β-galactosidase activity profiles associated with the two H3-T6SS transcriptional fusions were similar. Expression was induced at the transition from log-to-stationary phase and a maximal level was reached late in stationary phase, after 9 hours of growth (Fig. 3A and Fig. S1). The expression then stayed and maximal for at least 4 more hours. No promoter activity was detectable in the control strain PAO1Z. Moreover, *H3-T6SS* was clearly more expressed at 37°C than at the other tested temperatures, 30°C and 42°C (Fig. S1). As previously observed [14], *H2-T6SS* expression was also induced at the transition phase, but a maximal and steady level of expression was reached earlier in stationary phase (Fig. 3A and Fig. S1).

The cell density dependent expression profile of the H3-T6SS reporter fusions suggested that the transcription of the *H3-T6SS* might be regulated by QS. This would be in line with the LasR –mediated regulation of *hcp3* in the H3-T6SS right operon of PA14 [17], and with two transcriptomic studies indicating de-regulated expression of genes in the right H3-T6SS operon of PAO1 in QS mutants [22], [23]. We therefore examined the expression of the two H3-T6SS fusions in *P. aeruginosa* QS mutants, a *lasR* mutant (PAOR) [24] and a *rhlI* mutant (PDO100) [25]. Compared to the WT PAO1 strain (Fig. 3B & 3C), the expression of the left and right H3-T6SS operons was significantly decreased in the *lasR* mutant (2.7-fold and 3.3-fold respectively) and in the *rhlI* mutant (2.4-fold and 2.7-fold respectively). In conclusion, *H3-T6SS* is coregulated with *H2-T6SS* by the Las and Rhl QS systems.

### Divergent Effects of RpoN on H2- and H3-T6SS Gene Expression

Several reports in the literature have shown that T6SS transcriptional activity requires the sigma factor RpoN (σ54) and cognate activators encoded within the T6SS operon. In the initial genetic screen that led to the discovery of T6SS genes in *V. cholerae* [2], one of the attenuated mutants in virulence towards *Dictyostelium* was in the *vasH* gene which encodes a σ54-activator. Similarly, in *Aeromonas hydrophila*, a VasH homologue was shown to be required for cytotoxicity towards macrophages and epithelial cells since it was found to be essential for the expression of the genes encoding the T6SS machinery [26]. In agreement, Bernard and coworkers demonstrated, in a reconstituted heterologous system, that σ54-activators from various T6SSs together with the *E. coli* σ54-RNAP (RNA polymerase) complex allowed expression of T6SS genes [27].

**Figure 4:**
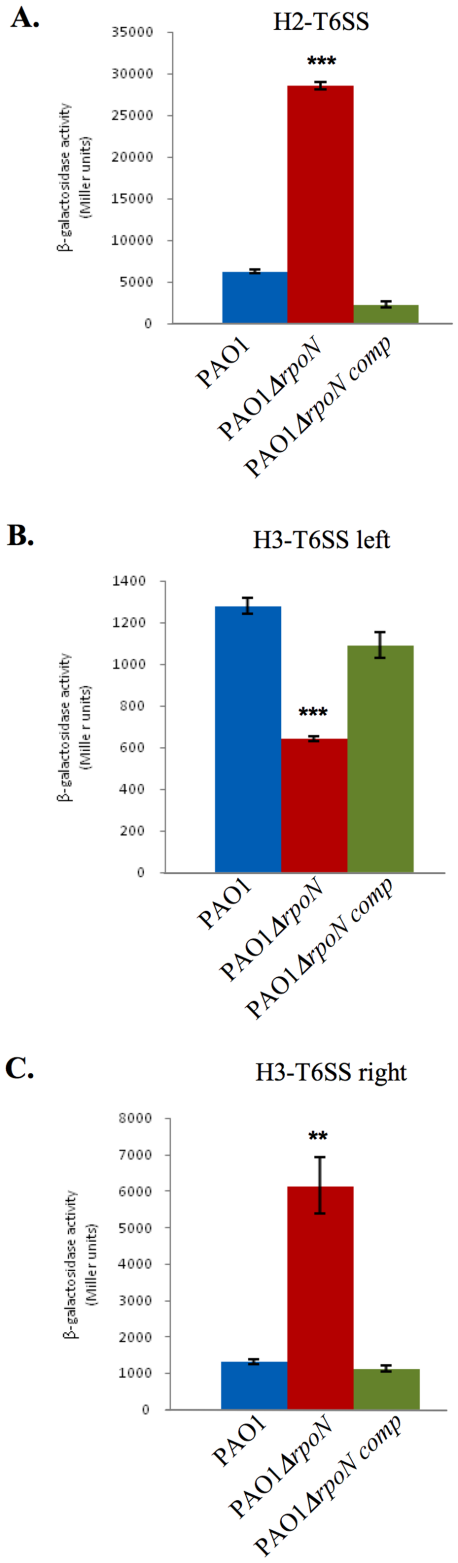
Divergent control of RpoN on *H2-T6SS* and *H3-T6SS*. The expression of *H2-T6SS* **(A)**, *H3-T6SS* left **(B)**, and *H3-T6SS* right **(C)** after 7 hours **(A)**, or 9 hours **(B & C)** of growth at 37°C in the WT strain (blue bars), in a PAO1Δ*rpoN* mutant complemented strain (green bars, PAO6360 strain) or the PAO1 Δ*rpoN* mutant (red bars, PAO6358 strain). Expression is given in Miller units. Each experiment was done in triplicate and independently repeated three times; error bars indicate the standard deviation.

We thus monitored expression of the H2-T6SS and H3-T6SS *lacZ* fusions in the *rpoN* mutant (Table 1) at the entry to stationary phase (Fig. 4). The *rpoN* mutation is complemented (PAO6360) or not (PAO6358) on the chromosome [28]. Expression of the left H3-T6SS operon decreased 2.0-fold in the *rpoN* mutant, and complementation of the mutation restored WT expression levels (Fig. 4B). This means that expression of the left H3-T6SS operon depends on RpoN, and may require a σ54-activator as observed in for other T6SSs. Unexpectedly, H2-T6SS and the right H3-T6SS operons were overexpressed in the *rpoN* mutant (4.5-fold and 4.7-fold increase respectively). Expression was restored to WT levels upon complementation (Fig. 4A & 4C). This suggests that RpoN mediates the transcriptional repression of these two operons. In conclusion, RpoN has divergent roles in regulating *P. aeruginosa* T6SS gene expression.

### Sfa2 Decreases *H2-T6SS* Expression whereas *H3-T6SS* Expression is Sfa-independent

**Figure 5:**
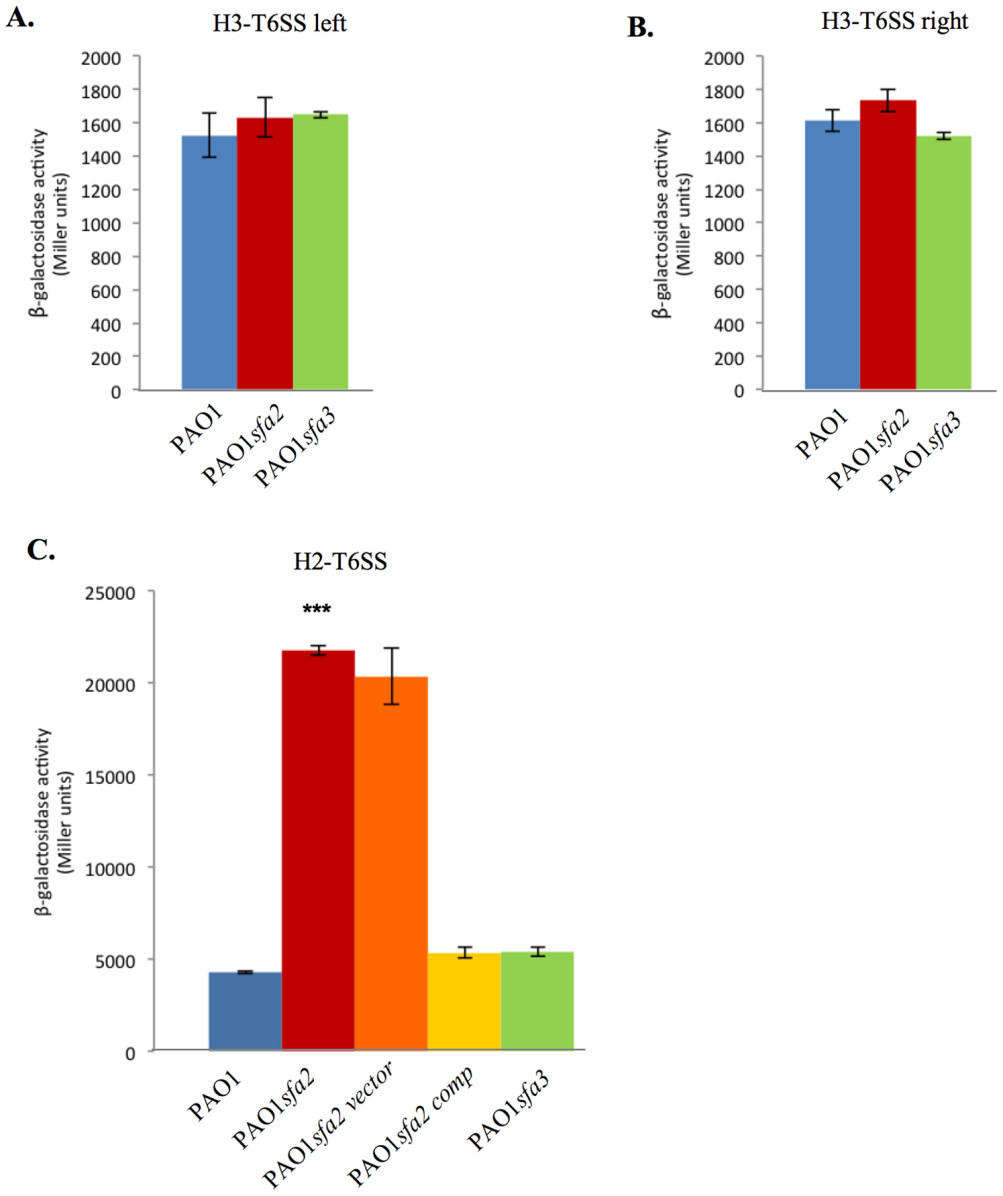
Sfa2 negatively controls *H2-T6SS* expression while *H3-T6SS* expression is Sfa-independent. The expression of *H3-T6SS* left **(A)**, *H3-T6SS* right **(B)**, and *H2-T6SS* **(C)** after 6 hours of growth after 9 hours **(A & B)** or 7 hours **(C)** at 37°C in the WT strain (blue bars), in a PAO1*sfa2* mutant (red bars), and in a PAO1*sfa3* mutant (green bars). Expression is given in Miller units. Each experiment was done in triplicate and independently repeated three times; error bars indicate the standard deviation.

In *P. aeruginosa*, the *sfa2* and *sfa3* genes (sigma factor activator) from the H2- and H3-T6SS loci (Fig. 2A & 2B) encode putative RpoN activators also called EBPs (enhancer binding protein) [7]. As for other EBPs, Sfa2 and Sfa3 contain two Walker A and B motifs that have roles in nucleotide binding and hydrolysis, and the highly conserved “GAFTGA” domain that is indispensable for the nucleotide-dependent interactions with σ54-RNAP that drives open complex formation and transcription (Fig. S2) [29], [30]. We thus wondered if Sfa2 and Sfa3 proteins could be involved in H2- and H3-T6SS regulation. To test this hypothesis, *sfa2* and *sfa3* mutants were constructed and expression of the three *lacZ* fusions were assayed in the mutant backgrounds. While mutations in *sfa2* and *sfa3* had no effect on H3-T6SS expression levels (Fig. 5A & 5B), H2-T6SS expression was increased in the *sfa2* mutant (3.7-fold). Expression could be restored to WT levels upon complementation *in trans* with a WT copy of *sfa2* (Fig. 5C). Moreover Sfa3 has no effect on *H2-T6SS* expression (Fig. 5C). Taken together, these data suggest that *H3-T6SS* expression is Sfa-independent and that the H2-T6SS operon may be indirectly repressed by RpoN in a Sfa2-dependent manner.

### Sfa2 Contributes to Repression of *H2-T6SS* by RpoN

**Figure 6:**
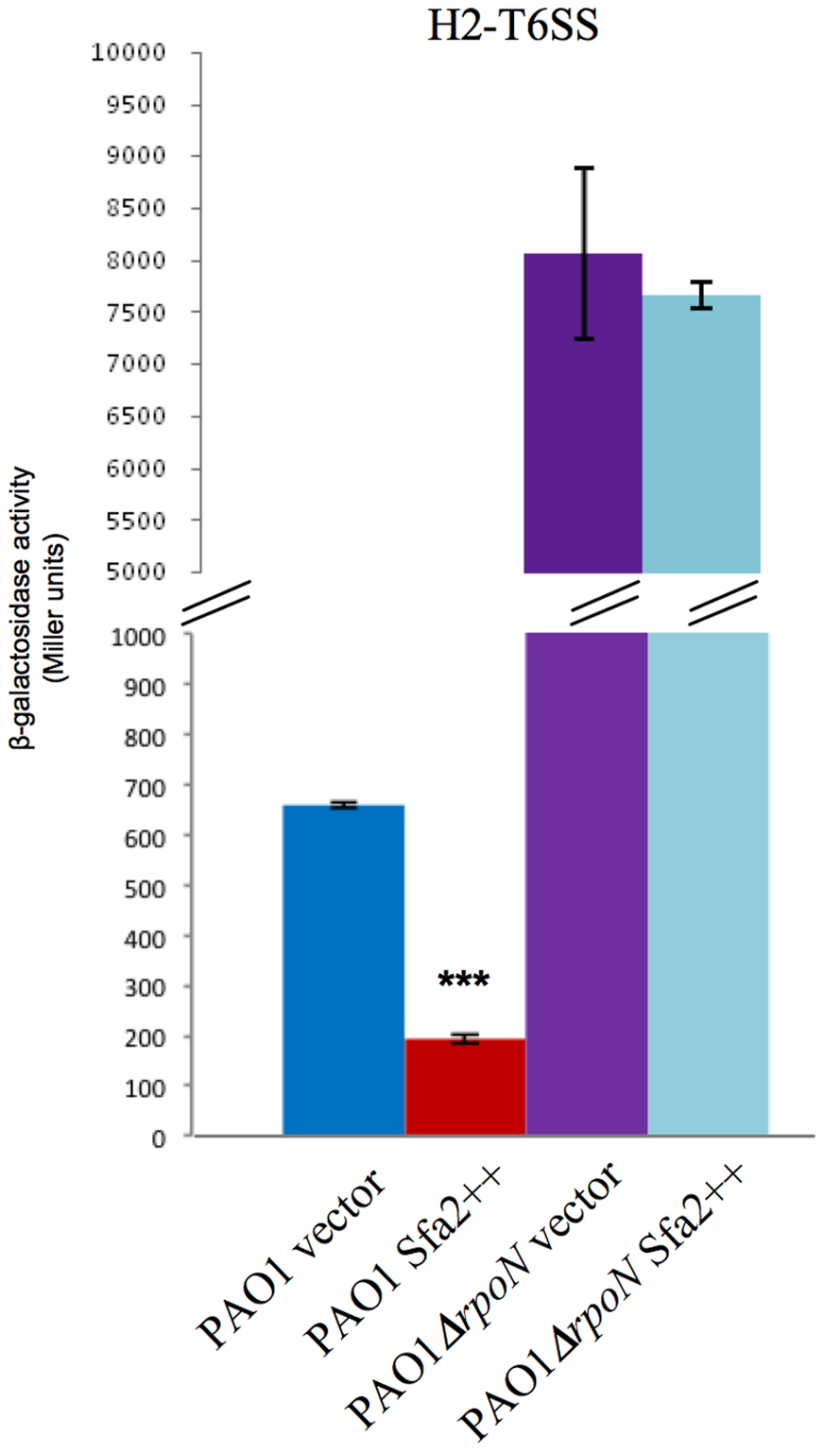
Sfa2 mediates down-regulation of *H2-T6SS* by RpoN. Expression of *H2-T6SS* after 3.5 hours of growth at 37°C in WT (blue and red bars) or *rpoN* mutant (purple and light blue bars) strains overproducing Sfa2 *in trans* (red or light blue bars), or without Sfa2 overproduction (empty vector, blue or purple bars). Expression is given in Miller units. For Sfa2 overproduction gene expression from the P_BAD_ promoter was induced with arabinose (0.5%). Each experiment was done in triplicate and independently repeated three times; error bars indicate the standard deviation.

To demonstrate that the RpoN repression of *H2-T6SS* is mediated by Sfa2, we hypothesized that overproduction of Sfa2 should have no effect in a *rpoN* mutant while it should decrease expression in a WT background. We thus monitored *H2-T6SS* expression upon Sfa2 overproduction in exponential phase in these two backgrounds. We chose to probe expression at this particular moment of growth to focus on the effect of the overproduced Sfa2 and not of the chromosomal *sfa2* gene, which not yet fully expressed at this time. In support of our hypothesis we indeed found that Sfa2 overproduction led to the decreased expression of *H2-T6SS* in the WT, and had no effect in the *rpoN* background (Fig. 6). Hence Sfa2 mediates to the indirect repression of *H2-T6SS* by RpoN.

## Discussion

The *P. aeruginosa* genome harbors three T6SS clusters. The H1-T6SS of the PAO1 strain targets toxins into host bacteria [8], [13] and H2-T6SS is involved in interactions with eukaryotic and prokaryotic hosts [14], [15]. The role of H3-T6SS has been mainly studied in the PA14 strain of *P. aeruginosa*, which appears to differ from PAO1 at the H2- and H3-T6SS gene level. The PA14 H3-T6SS is required for virulence in the plant model *A. thaliana* and may compensate for the loss of H2-T6SS in mouse virulence because only the double H2-T6SS H3-T6SS mutant is less virulent than the WT strain [17]. Here, we have shown that H3-T6SS is required for *P. aeruginosa* PAO1 virulence towards worms. However, we did not observe any compensation between the two T6SSs, at least in the worm model. But the *clpV3* mutation phenotype is dominant on *clpV2* since the double mutant has the same virulence defect as the *clpV3* mutant this suggesting a genetic interaction. Moreover, we found that RpoN divergently regulates expression of PAO1 T6SSs, by repressing *H2-T6SS* and the right *H3-T6SS* and activating the left *H3-T6SS*. Interestingly solely Sfa2, the σ54 activator encoded within the H2-T6SS cluster, participates in RpoN-control. This is the first example of a RpoN-repression mechanism mediated by a T6SS-encoded activator.

We observed that like *H2-T6SS* [14], the two H3-T6SS operons of the PAO1 strain are induced at the growth phase transition by LasR and RhlR QS regulators (Fig. 3). This is in line with data establishing a differential regulation of the three PA14 T6SS loci by LasR [17], [31]. We also noticed that the two H3-T6SS operons are fully expressed late in stationary phase (Fig. 3A), suggesting induction by the RpoS sigma factor. In agreement, a transcriptomic study showed that the right H3-T6SS operon is strongly dependent on RpoS [32], however the authors did not identify the heptameric consensus sequence CTATACT defined as the - 10 of RpoS-controlled promoters [33] upstream of the right H3-T6SS operon. Interestingly, the right H3-T6SS operon also belongs to a group of genes that are induced by contact with eukaryotic cells [34]. This is in agreement with our data on the involvement of H3-T6SS in *P. aeruginosa* virulence in a eukaryotic model (Fig. 1). Furthermore, we also observed that *H3-T6SS* is more highly expressed at 37°C, the temperature of certain eukaryotic hosts (Fig. S1). Finally, the H3-T6SS from PAO1 was recently shown to be dispensable for bacterial competition (Russell 2013). All together, and in contrast to H2-T6SS, the H3-T6SS machinery in PAO1 appears to be exclusively dedicated to interactions with eukaryotic cells.

We also showed a complex RpoN-control of T6SS expression. Indeed H2-T6SS and the right H3-T6SS operons are unexpectedly under a negative control by RpoN, to the contrary of other T6SSs (for a review see [5]), (Fig. 4A & 4C). Moreover, the RpoN-mediated repression of *H2-T6SS* requires Sfa2, the EBP encoded within H2-T6SS (Fig. 5C & 6). H3-T6SS right is Sfa-independent, although H3-T6SS left encodes an EBP, Sfa3 (Fig. 5B). We also showed that the H3-T6SS left operon is activated by RpoN, and independently of any Sfa (Fig. 5A). Hence the two H3-T6SS operons are divergently regulated by RpoN. This could fit with the recent observations of Dong and Mekalanos [35]. In *V. cholerae*, they observed that RpoN positively regulates the expression of the *hcp* operons and *vgrG3* that encode Type VI secreted proteins, but has no effect on the expression of the main T6SS cluster encoding sheath and other structural components of the phage tail-like machinery [5], [36]. This is presumably because the latter are recycled. The role of Sfa3 is also intriguing. It may be required for coregulation of substrate genes that are not part of the H3-T6SS locus.

In *P. aeruginosa*, RpoN plays important roles in mobility, in the transport of nutrients, in the formation of pili, in mucoidy and in cell-to-cell signaling (for a review see [37]). As a sigma factor, RpoN positively regulates its target genes (e.g. type IV pili and flagellum genes), but has also been shown to negatively regulate QS [28], the expression of *sadB*, coding an important protein during biofilm formation [38], and of *aceA*, coding an isocitrate lyase, an enzyme required for the metabolic pathway utilized by *P. aeruginosa* during chronic pulmonary infections [39]. Unlike RpoN-mediated activation, RpoN repression is indirect. In agreement we have been unable to identify RpoN consensus elements in the promoter regions of H2- and of the right H3-T6SS operons with Virtual footprint (http://www.prodoric.de/vfp/vfp_promoter.php), while a RpoN binding site was previously proposed for the H3 left operon that we found RpoN-activated [27] (Bernard 2011).

Taken together, our data allow us to propose a model in which the expression of *H2-T6SS* and *H3-T6SS* is induced by QS [14] at the transition between the exponential and stationary phase, with *H3-T6SS* reaching full expression later than *H2-T6SS* in the stationary phase. The H2-T6SS operon codes Sfa2, that once produced activates RpoN to repress its own expression. A first explanation in line with Heulier *et al*. [28] could have been that Sfa2 is the EBP which activates RpoN in order to repress QS, thus arresting *H2-T6SS* induction. However, we found that *lasR* and *rhlR* transcriptional *lacZ* fusions were not impacted upon Sfa2 overproduction (Fig. S3). We would thus like to propose that RpoN together with Sfa2 activates an unknown repressor of *H2-T6SS*. Repression of the H3-T6SS right operon might be explained by the RpoN/GacA/RsmA pathway [40]. Indeed, the H3-T6SS right operon is strongly activated by GacA and RsmYZ [41], while RpoN has a negative effect on *gacA* expression [28] and thus on expression of *H3-T6SS* right. Future studies will be required to decipher this network of regulation.

## Supporting Information

Figure S1 ***H2-T6SS*** **and** ***H3-T6SS*** **are differentially thermoregulated.** The expression pattern of the *H2-T6SS-lacZ* (A), *H3-T6SS left-lacZ* (B), and *H3-T6SS right-lacZ* (C) transcriptional fusions in the WT PAO1 strain is given in Miller Units at different time points over the growth and at 3 different temperatures: 30°C (blue), 37°C (green) and 42°C (red). The OD_600_ is also presented (diamonds). A control strain (PAO1Z) (grey squares) is included for each graph. Each experiment was done in triplicate and independently repeated three times; error bars indicate the standard deviation.
(TIF)

Figure S2 **Sfa2 and Sfa3 are EBPs. Sfa2 (A) and Sfa3 (B) are 503 and 361 amino acids long.** Both proteins possess WalkerA, Switch ASN, GAFTGA, WalkerB and Arg Fingers motifs that are specific to σ^54^ activators.
(TIF)

Figure S3 **Sfa3 has no effect on QS gene expression.** Expression of *rhlR-lacZ* (A) and *lasR-lacZ* (B) transcriptional fusions is given in Miller Units after 4 h of growth in the PAO1 strain overproducing Sfa2 (red bars) or not (blue bars, empty vector). Each experiment was done in triplicate and independently repeated three times; error bars indicate the standard deviation.
(TIF)
