# Supplementary figures and images for: Divergent Control of Two Type VI Secretion Systems by RpoN in Pseudomonas aeruginosa
Source: PLoS One. 2013 Oct 21;8(10):e76030. doi: 10.1371/journal.pone.0076030 (PMC3804575; doi:10.1371/journal.pone.0076030)

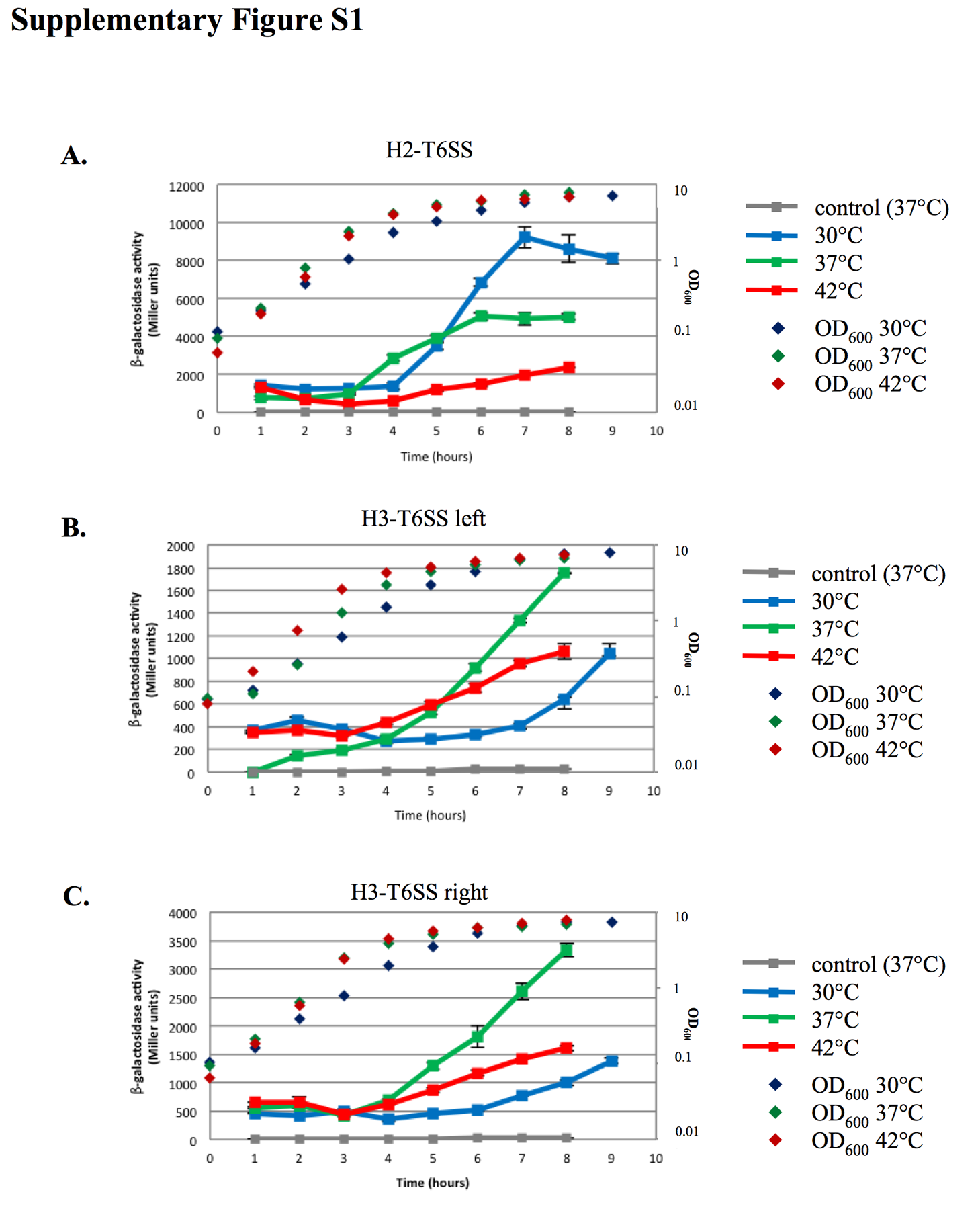

Supplement: Figure S1 — H2-T6SS and H3-T6SS are differentially thermoregulated. The expression pattern of the H2-T6SS-lacZ (A), H3-T6SS left-lacZ (B), and H3-T6SS right-lacZ (C) transcriptional fusions in the WT PAO1 strain is given in Miller Units at different time points over the growth and at 3 different temperatures: 30°C (blue), 37°C (green) and 42°C (red). The OD600 is also presented (diamonds). A control strain (PAO1Z) (grey squares) is included for each graph. Each experiment was done in triplicate and independently repeated three times; error bars indicate the standard deviation. (TIF) [file pone.0076030.s001.tif]

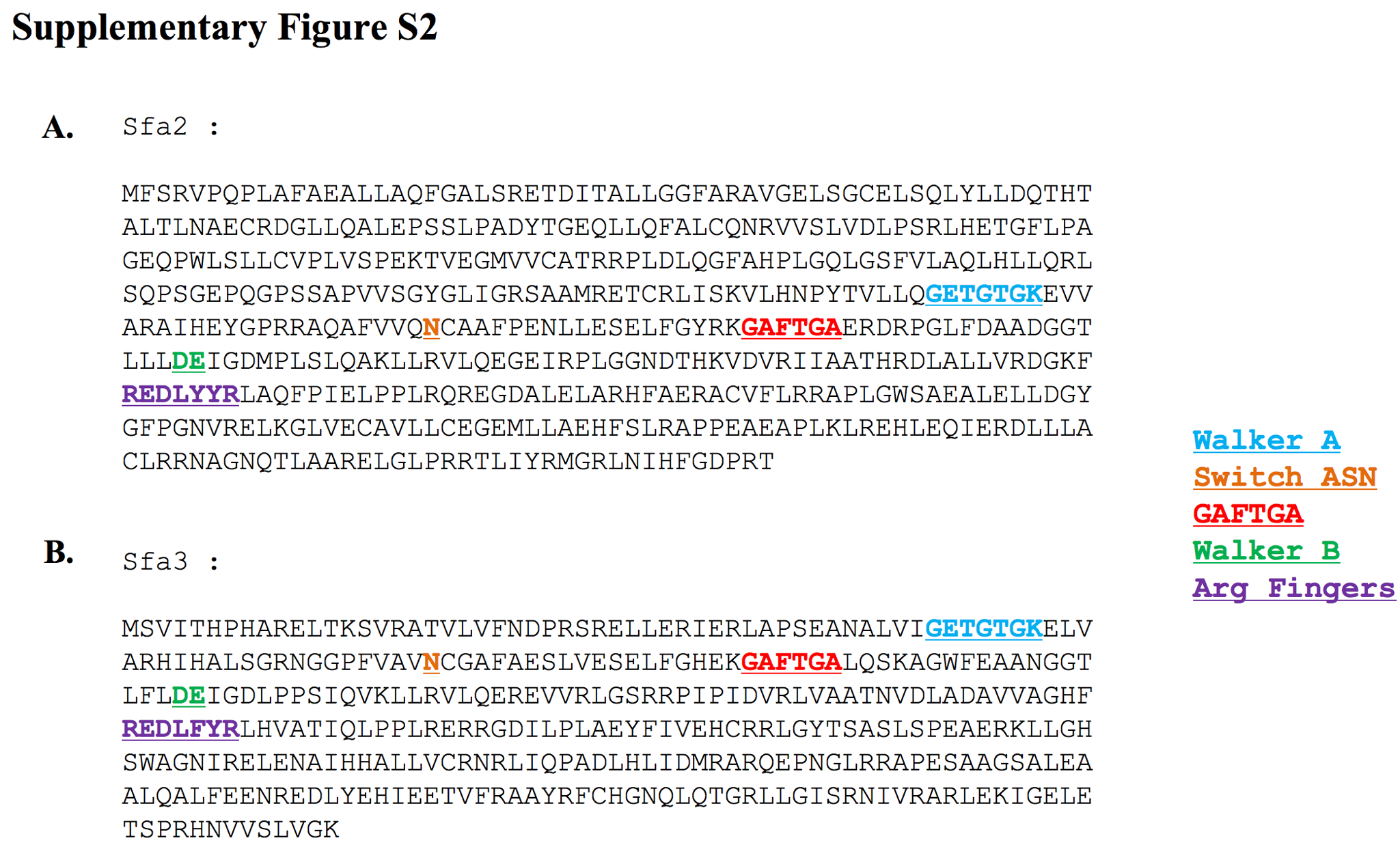

Supplement: Figure S2 — Sfa2 and Sfa3 are EBPs. Sfa2 (A) and Sfa3 (B) are 503 and 361 amino acids long. Both proteins possess WalkerA, Switch ASN, GAFTGA, WalkerB and Arg Fingers motifs that are specific to σ54 activators. (TIF) [file pone.0076030.s002.tif]

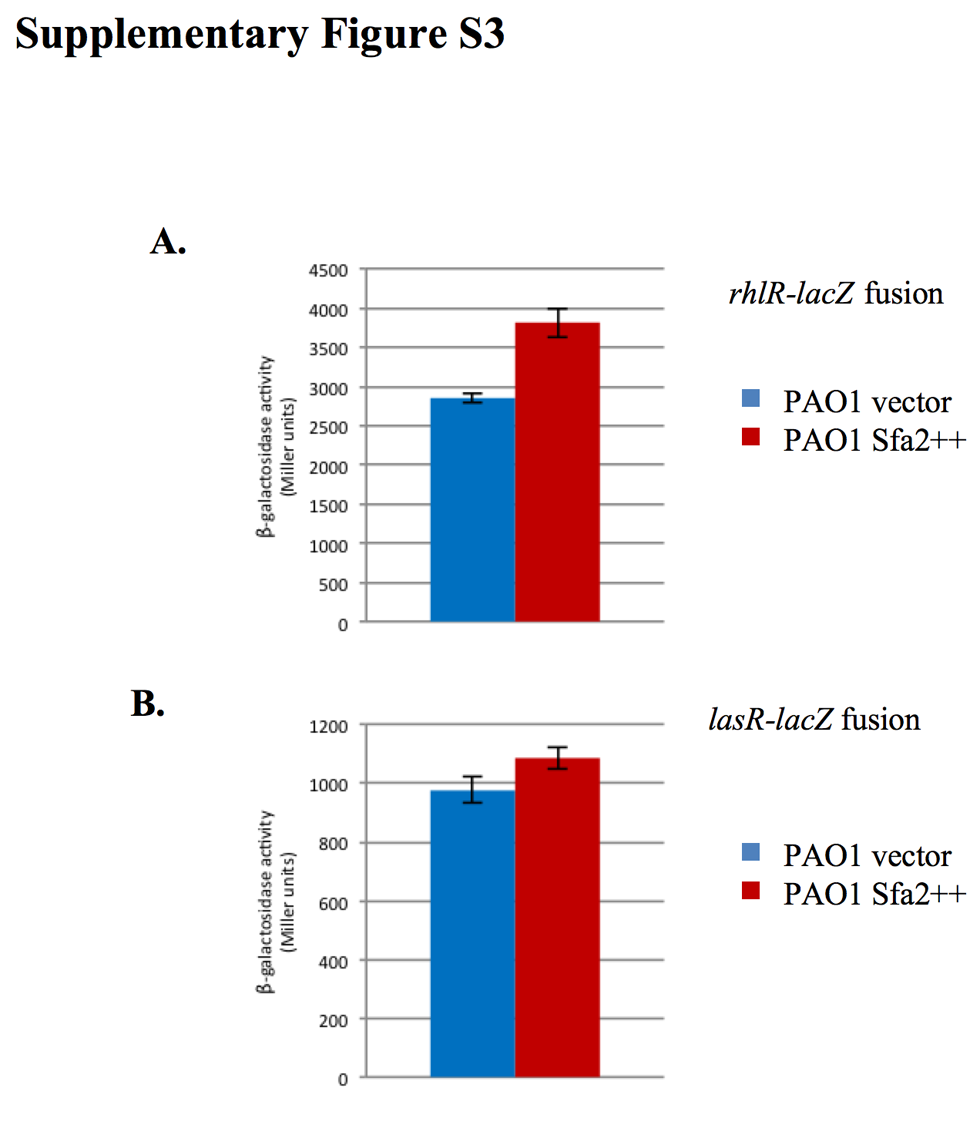

Supplement: Figure S3 — Sfa3 has no effect on QS gene expression. Expression of rhlR-lacZ (A) and lasR-lacZ (B) transcriptional fusions is given in Miller Units after 4 h of growth in the PAO1 strain overproducing Sfa2 (red bars) or not (blue bars, empty vector). Each experiment was done in triplicate and independently repeated three times; error bars indicate the standard deviation. (TIF) [file pone.0076030.s003.tif]
